# Increase in coleoptile length and establishment by *Lcol-A1*, a genetic locus with major effect in wheat

**DOI:** 10.1186/s12870-019-1919-3

**Published:** 2019-07-29

**Authors:** William D. Bovill, Jessica Hyles, Alexander B. Zwart, Brett A. Ford, Geetha Perera, Tanya Phongkham, Brenton J. Brooks, Gregory J. Rebetzke, Matthew J. Hayden, James R. Hunt, Wolfgang Spielmeyer

**Affiliations:** 1 CSIRO Agriculture and Food, P.O. Box 1700, Canberra, ACT 2601 Australia; 2 Data61, CSIRO, P.O. Box 1700, Canberra, ACT 2601 Australia; 3 Agriculture Victoria Research, AgriBio Centre for AgriBiosciences, Bundoora, VIC 3086 Australia; 4 Department of Animal, Plant and Soil Sciences, AgriBio Centre for AgriBiosciences, La Trobe University, Bundoora, VIC 3086 Australia

**Keywords:** *Triticum aestivum*, Wheat, Coleoptile, Emergence, Molecular marker, SNP

## Abstract

**Background:**

Good establishment is important for rapid leaf area development in wheat crops. Poor establishment results in fewer, later-emerging plants, reduced leaf area and tiller number. In addition, poorly established crops suffer from increased soil moisture loss through evaporation and greater competition from weeds while fewer spikes are produced which can reduce grain yield. By protecting the emerging first leaf, the coleoptile is critical for achieving good establishment, and its length and interaction with soil physical properties determine the ability of a cultivar to emerge from depth.

**Results:**

Here we characterise a locus on chromosome 1AS, that increases coleoptile length in wheat, which we designate as *Lcol-A1.* We identified *Lcol-A1* by bulked-segregant analysis and used a Halberd-derived population to fine map the gene to a 2 cM region, equivalent to 7 Mb on the IWGSC genome reference sequence of Chinese Spring (RefSeqv1.0). By sowing recently released cultivars and near-isogenic lines in the field at both conventional and deep sowing depths, we confirmed that *Locl-A1* was associated with increased emergence from depth in the presence and absence of conventional dwarfing genes. Flanking markers *IWB58229* and *IWA710* were developed to assist breeders to select for long coleoptile wheats.

**Conclusions:**

Increased coleoptile length is sought in many global wheat production areas to improve crop emergence. The identification of the gene *Lcol-A1*, together with tools to allow wheat breeders to track the gene, will enable improvements to be made for this important trait.

**Electronic supplementary material:**

The online version of this article (10.1186/s12870-019-1919-3) contains supplementary material, which is available to authorized users.

## Background

In water-limited environments with high evaporative demand, wheat seedlings need to emerge and develop leaf area rapidly to allow good establishment. Poor establishment reduces the number of spikes per square meter and grain yield [32, 37]. By protecting the emerging first leaf, the coleoptile is critical for achieving good establishment, and its length and interaction with soil physical properties determine the ability of a cultivar to emerge from depth [11, 12, 30].

Longer coleoptiles are beneficial in many agricultural systems worldwide, particularly where deep sowing into moisture is required [20, 38, 44], where stubble retention practices have been adopted [34] or where crops are sown early into warmer soils [7, 36]. In Australia, there exists a narrow period during which crops must flower in order for yields to be maximised [18], and crop yield is thus very sensitive to timing of establishment. There is an emerging trend to sow crops earlier into warmer soils to optimise whole-farm logistics and increase water-use efficiency [16, 17, 19, 28], but seed-bed soil water potentials are often sub-optimal and temperatures supra-optimal for germination and emergence at this time. The lack of surface moisture during the short, optimum sowing window encourages farmers to sow deep, but if the coleoptile is short, the first leaf may not emerge or may be damaged, leading to poor establishment [38, 44]. Many current cultivars are not be suited to deep sowing due to their short coleoptiles, and warm soils exacerbate this by further shortening coleoptile length [36]. Long coleoptile wheats would provide growers with more flexibility to sow deep, but at present a lack of knowledge about genes that promote coleoptile growth and efficient selection tools prevent breeders from incorporating this important trait.

Most semi-dwarf cultivars grown worldwide have relatively short coleoptiles due to the presence of dwarfing genes *Rht-B1b* and *Rht-D1b* [23]. These genes encode mutant DELLA proteins that are negative regulators of growth with a large negative effect on coleoptile length which is associated with poor establishment in the field [1, 2, 15, 42]. Despite this, several studies have found variation for coleoptile length within semi-dwarf wheats, suggesting that loci which increase coleoptile length can be selected for in semi-dwarf backgrounds [1, 5].

The search for genetic variation of increased coleoptile length has progressed from phenotypic screening to genome-wide approaches including QTL mapping and genome-wide association studies (GWAS). Preliminary QTL studies focussed on interval mapping approaches in bi-parental populations with sparse maps constructed using AFLP or microsatellite markers [27, 31, 33, 40, 41, 45]. This approach identified multiple QTL with generally small effect (explaining < 5% of phenotypic variation) and large confidence intervals. The advent of cost-effective high density SNP markers and more complex population structures has enabled greater mapping resolution. Rebetzke et al. [35], using a 4-way multi-parent advanced generation intercross (MAGIC) wheat population genotyped with the 9 K SNP array [10], identified seven QTL, while GWAS on a set of 893 wheat accessions also genotyped with the 9 K SNP array identified eight QTL for coleoptile length [26]. Both mapping studies identified QTL on either or both of chromosomes 4B and 4D associated with the dwarfing genes *Rht-B1b* and *Rht-D1b*, confirming the negative effect of these genes on coleoptile length, but also identified loci independent of the dwarfing genes which increased coleoptile length.

Previous studies highlight that coleoptile length is controlled by many genes with relatively small effects. This study reports the identification, fine mapping, and field validation of a coleoptile length-promoting gene on chromosome 1A of bread wheat. We show that the gene promotes coleoptile length in current elite germplasm, and that longer coleoptiles result in better emergence in both tall and semi-dwarf wheat lines when sown deep in the field.

## Results

### Genomic regions associated with long coleoptiles

**Fig. 1:**
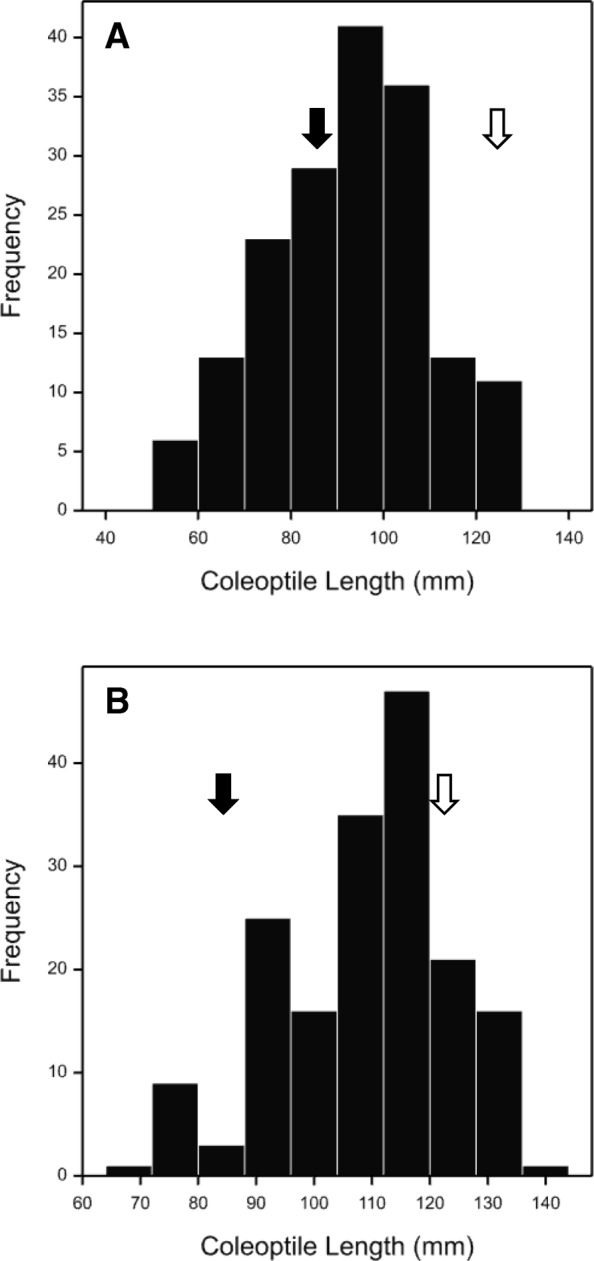
Phenotypic distribution of coleoptile length in 188 BC_1_F_2_ lines from each of the Halberd*2/CM18 population (**a**) and the Uruguay386*2/CM18 populations (**b**). Parental means are indicated by filled (CM18) and unfilled (Halberd and Uruguay) arrows

Halberd and Uruguay386 are tall wheats known for their long coleoptiles. To identify genomic regions contributing to the trait, the coleoptile length was measured in BC_1_F_2_ lines from two backcross-derived populations of Halberd or Uruguay386 to the short coleoptile Chinese wheat line CM18. As expected, Halberd and Uruguay displayed a long coleoptile phenotype and CM18 a short coleoptile phenotype (Fig. 1). Lines with extremely long and short coleoptiles were selected from these populations for bulked-segregant analysis (BSA) using the 9 K wheat SNP array [10]. Marker-trait associations (MTAs) were detected on 13 wheat chromosomes in the Halberd*2/CM18 population, and 8 chromosomes in the Uruguay*2/CM18 population (Additional file 1: Table S1). Because a large number of tightly linked SNP markers on chromosome 1A were strongly associated with the trait in both populations (Table 1), it was decided to genetically map the trait in this region.

**Table 1:** Significant (*p* < 0.0001) SNPs on chromosome 1A detected from the bulked-segregant analysis (BSA) in the Halberd*2/CM18 and Uruguay386*2/CM18 populations. Locations (_Loc; cM) from the 9 K [10] and 90 K [43] consensus maps are provided. NA = SNP not placed on consensus map

SNP Name	SNP ID	9K_Loc	90K_Loc
Halberd*2/CM18			
wsnp_Ku_c17726_26872129	IWA6636	51.12	71.05
wsnp_Ex_c3253_5995011	IWA3399	54.28	NA
wsnp_Ex_c6826_11774795	IWA4578	57.95	70.10
wsnp_Ku_c3468_6420199	IWA6942	57.95	70.10
wsnp_Ex_c3747_6824863	IWA3612	57.95	70.10
wsnp_Ex_c8364_14095508	IWA4797	62.39	71.10
wsnp_Ku_c37925_46679146	IWA6972	70.70	71.10
wsnp_Ku_c33917_43336069	IWA6934	109.32	101.19
Uruguay386*2/CM18			
wsnp_Ex_c5323_9408829	IWA4163	35.82	51.09
wsnp_Ku_c11896_19337444	IWA6441	39.00	NA
wsnp_BE586140A_Ta_2_1	IWA360	40.65	55.18
wsnp_Ex_c34821_43076533	IWA3499	51.12	71.48
wsnp_Ex_c3253_5995011	IWA3339	54.28	NA
wsnp_Ex_c2749_5091813	IWA3115	56.97	70.10
wsnp_JD_rep_c49359_33578909	IWA6260.1	57.95	70.10
wsnp_Ex_rep_c104050_88861052	IWA5109	57.95	70.10
wsnp_Ra_c26956_36503468	IWA7804	57.95	70.10
wsnp_Ex_c2389_4477096	IWA2847	57.95	70.10
wsnp_Ex_c3142_5808330	IWA3338	64.39	70.10
wsnp_BE518393A_Td_2_3	IWA352	64.39	70.10
wsnp_Ex_rep_c66382_64577768	IWA5226	67.01	70.10
wsnp_CAP11_c1029_611774	IWA639	68.32	70.10
wsnp_Ku_rep_c71909_71634013	IWA7505	68.65	70.79

### Fine-mapping of the 1A long coleoptile region

**Fig. 2:**
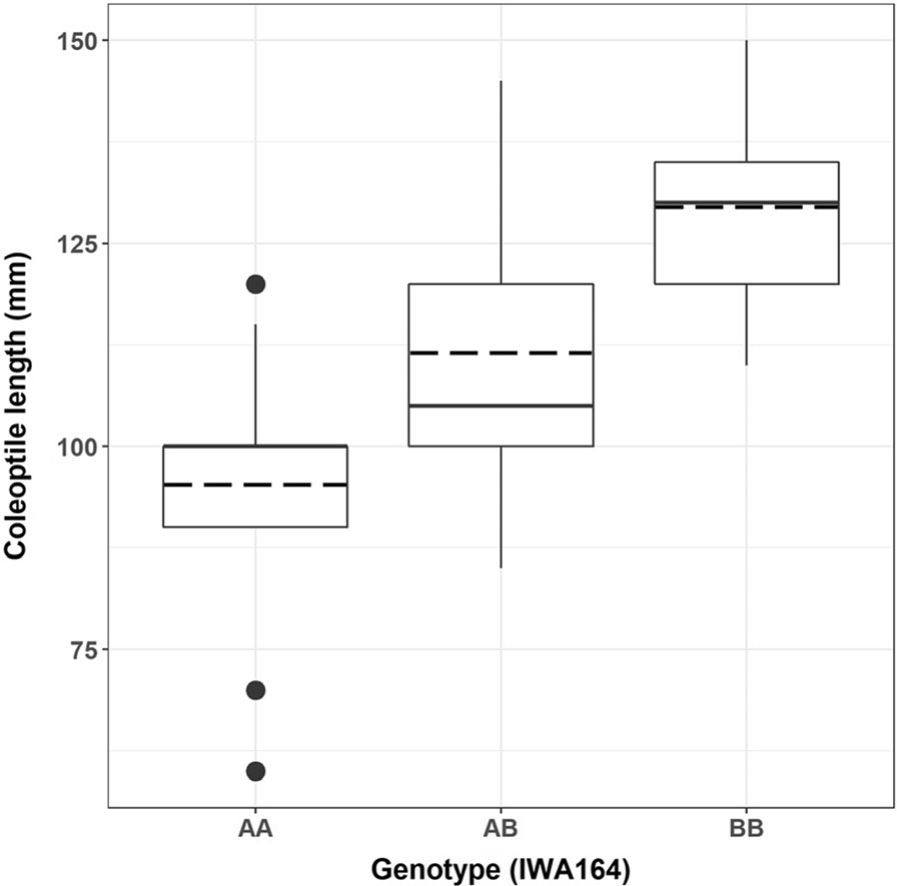
Association between genotype at marker *IWA164* and coleoptile length of BC_1_F_3_ seedlings from the Young*2/H125M. AA, homozygous negative; AB, heterozygous; BB homozygous positive. The lower and upper edges of the box represent 25th and 75th percentiles, interior solid lines denote the median and dashed lines denote the mean. Potential outliers are plotted as black dots, and identified according to the 1.5× interquartile range criterion

**Fig. 3:**
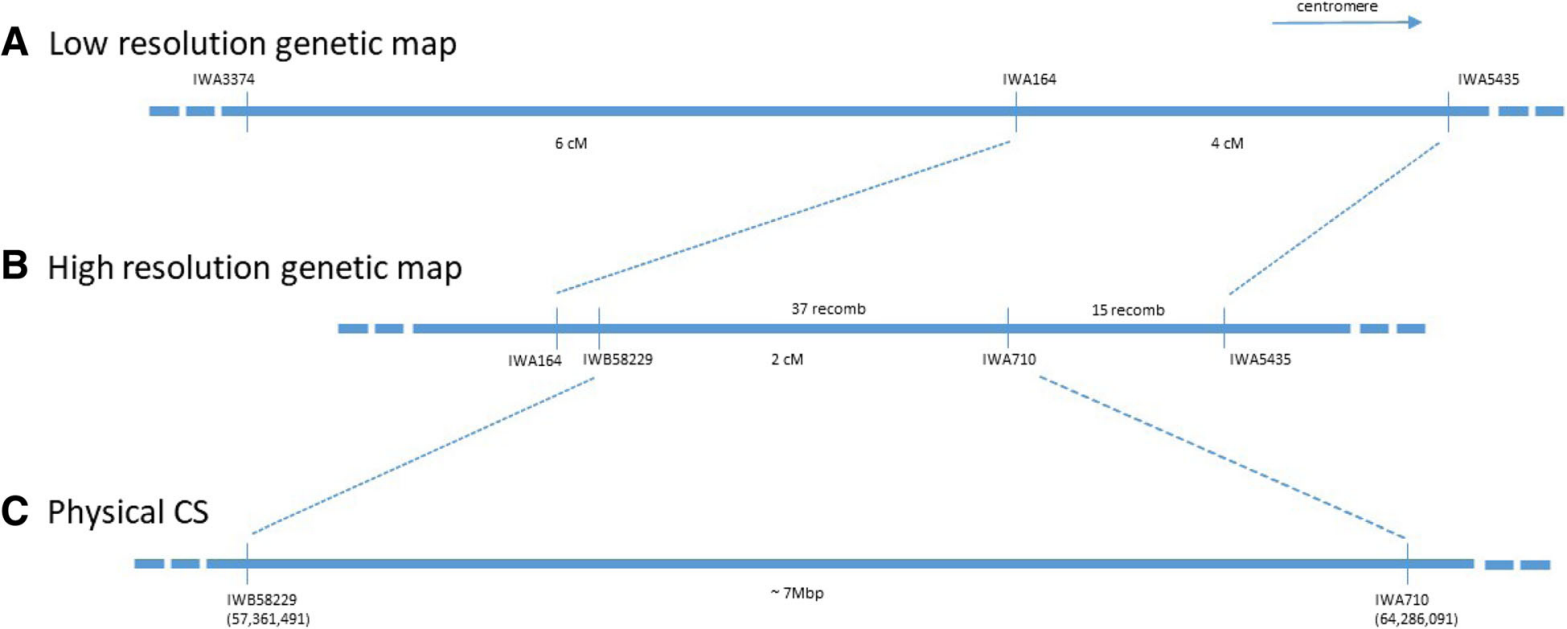
Genetic and physical maps of 1AS region carrying *Lcol-A1*. **a**: Low resolution genetic map based on 54 BC_1_F_2_:F_3_ lines of Young*2/H125M population, **b**: High resolution genetic map based on 900 BC_2_F_2_ equivalents of Young*3/H125M population, and **c**: corresponding physical map from Chinese Spring (CS; IWGSC RefSeqv1.0 genome sequence assembly)

For genetic mapping of the trait on chromosome 1A, a new backcross-derived population was developed from HI25M (a Halberd derivative) and Young that lack the *Rht-B1b* dwarfing gene. Fifty-four BC_1_F_2_ plants were genotyped with *IWA164*, a 1A marker selected from the 1A region in the 9 K SNP consensus map [10]. The association between marker *IWA164* and the trait was confirmed after BC_1_F_3_ families were phenotyped for coleoptile length, with the marker explaining approximately 30 mm of phenotypic variation for coleoptile length in this population (Fig. 2). Additional SNP-based markers *IWA3374* and *IWA5435* were selected from the 1A consensus map and shown to flank a genetic interval (~ 10 cM) which contained a gene that will be referred to as *Lcol-A1* (Fig. 3).

To refine the map location of *Lcol-A1*, flanking markers *IWA3374* and *IWA5435* were converted to KASP assays and used to screen 900 BC_2_F_2_ equivalents from the Young*3/HI25M population to identify 204 recombinants between *IWA3374* and *IWA5435*. These recombinants were genotyped with *IWA164* and a subset of 28 lines were selected for phenotyping. To improve phenotyping accuracy, recombination events were fixed by genotyping BC_2_F_3_ progeny and selecting segregants that were homozygous at flanking marker loci. Coleoptile length was measured on F_4_ families derived from homozygous recombinants to map *Lcol-A1* to a 4 cM interval flanked by markers *IWA164* and *IWA5435* (Fig. 3). An additional seven markers were added to the target interval by screening parental lines with the 90 K SNP array [43] and genotyping recombinants with polymorphic markers. In a second round of fine mapping, a further 57 recombinants (from total of 204) were selected based on *IWA164* and *IWA5435* marker genotypes to measure coleoptile length on BC_2_F_4_ homozygous, recombinant families. Consequently the map location of *Lcol-A1* was refined to the 2 cM marker interval between *IWB58229*-*IWA710* which corresponded to approximately 7 Mb of physical distance in the RefSeqv1.0 genome sequence assembly [3].

### Validation of Lcol-A1 in Espada genetic background

**Fig. 4:**
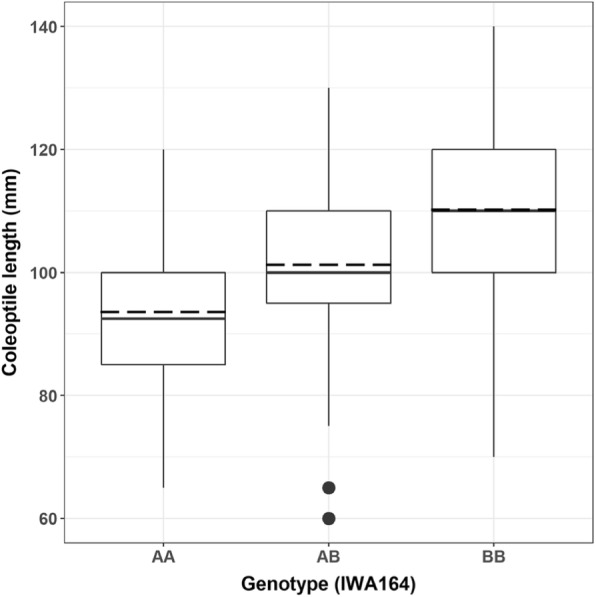
Association between genotype at marker *IWA164* and coleoptile length of BC_3_F_3_ seedlings from the Espada*4/H125M population. AA, homozygous negative; AB, heterozygous; BB homozygous positive. The lower and upper edges of the box represent 25th and 75th percentiles, interior solid lines denote the median and dashed lines denote the mean. Potential outliers are plotted as black dots, and identified according to the 1.5× interquartile range criterion

The effect of *Lcol-A1* was confirmed by transferring the long coleoptile haplotype from HI25M into the Australian cultivar Espada. In a backcross-derived population (Espada*4/HI25M) which lacked the *Rht-D1b* dwarfing gene from Espada, coleoptile length was continuously distributed ranging from 65 to 140 mm in length (Fig. 4). The same marker *IWA164* which was linked to the trait in the Young background explained approximately 25 mm of variation in homozygous lines carrying different haploytypes in the Espada background. These results confirm that *Lcol-A1* contributes to coleoptile growth in another genetic background indicating a major gene for coleoptile growth has been identified.

### Lcol-A1 contributes to better emergence in the field

To show that the long coleoptile trait conferred by *Lcol-1A* also improves plant emergence and establishment under field conditions, we tested the hypothesis that cultivars with *Lcol-A1* produce longer coleoptiles and emerge better when sown deep under field conditions compared to cultivars that lack the long coleoptile allele of the gene. Five cultivars that were predicted to carry the long allele based on the haplotype of flanking markers and pedigree and six that were predicted to carry the short allele were selected to test the hypothesis (Table 2). These released cultivars represented a wide range of genetic diversity of the Australian genepool. To determine the effect of *Lcol-A1* in tall background, isolines from the Young*3/HI25M population (five short and five long haplotype) were also evaluated for emergence under field conditions.

**Table 2:** Cultivars selected for examining the effect of *Lcol-A1* on emergence in the field. *Lcol-A1* haplotype and year of release (Year) are indicated*.* Pedigree information was retrieved from the Genetic Resources Information System for Wheat and Triticale (GRIS; wheatpedigree.net)

Cultivar	*Lcol-A1* Haplotype	Year	Pedigree
EGA Gregory	Short	2004	Pelsart/3*Batavia
Emu Rock	Short	2011	Westonia/Kukri/Perenjori/Ajana
Espada	Short	2008	RAC-875/Krichauff//Excalibur/Kukri/3/RAC-875/Krichauff/4/RAC-875//Excalibur/Kukri
Mace	Short	2008	Wyalkatchem/Stylet//Wyalkatchem
Suntop	Short	2011	Sunco/2*Pastor//SUN-436-E
Young	Short	2005	VPM-1/3*Beulah//Silverstar
Excalibur	Long	1990	RAC-177/Uniculm-492//RAC-311-S
Magenta	Long	2007	Carnamah/Tammin-18
Phantom	Long	2010	Sentinel*3/Yitpi
Scout	Long	2009	Sunstate/QH-76-1//Yitpi
Yitpi	Long	2000	C-8-MMC-8-HMM/Frame

Significant (p < 0.0001) SNPs on chromosome 1A detected from the bulked-segregant analysis (BSA) in the Halberd/CM18 and Uruguay386/CM18 backcross populations. Locations (_Loc; cM) from the 9 K [10] and 90 K [43] consensus maps are provided. NA = SNP not placed on consensus map

**Fig. 5:**
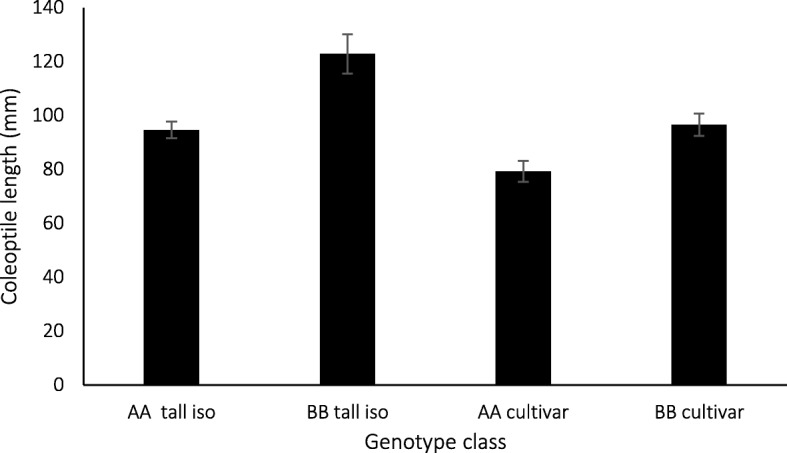
Coleoptile length of isolines (iso) and cultivars with long (BB) and short (AA) *Lcol-A1* alleles under controlled conditions. Data are means (*n* = 5 for each isoline class; 6 for the short [AA] cultivar class; and 5 for the long [BB] cultivar class, obtained by averaging values from varieties within the relevant [isoline vs cultivar] genotype class) ± standard error; differences within genotype class are significant (*p* = 0.011 for cultivars, *p* < 0.01 for tall isolines)

Prior to field testing, we confirmed that *Lcol-A1* contributed to coleoptile length in semi-dwarf cultivars which were included in field trials. The coleoptile length, when measured under controlled conditions, was approx. 18% longer in cultivars predicted to carry the long *Lcol-A1* allele (Fig. 5). In tall isolines the final length increased compared to the cultivars but the difference between genotypes also increased to approximately 25% consistent with an expected growth promotion effect in the absence of conventional dwarfing genes.

**Fig. 6:**
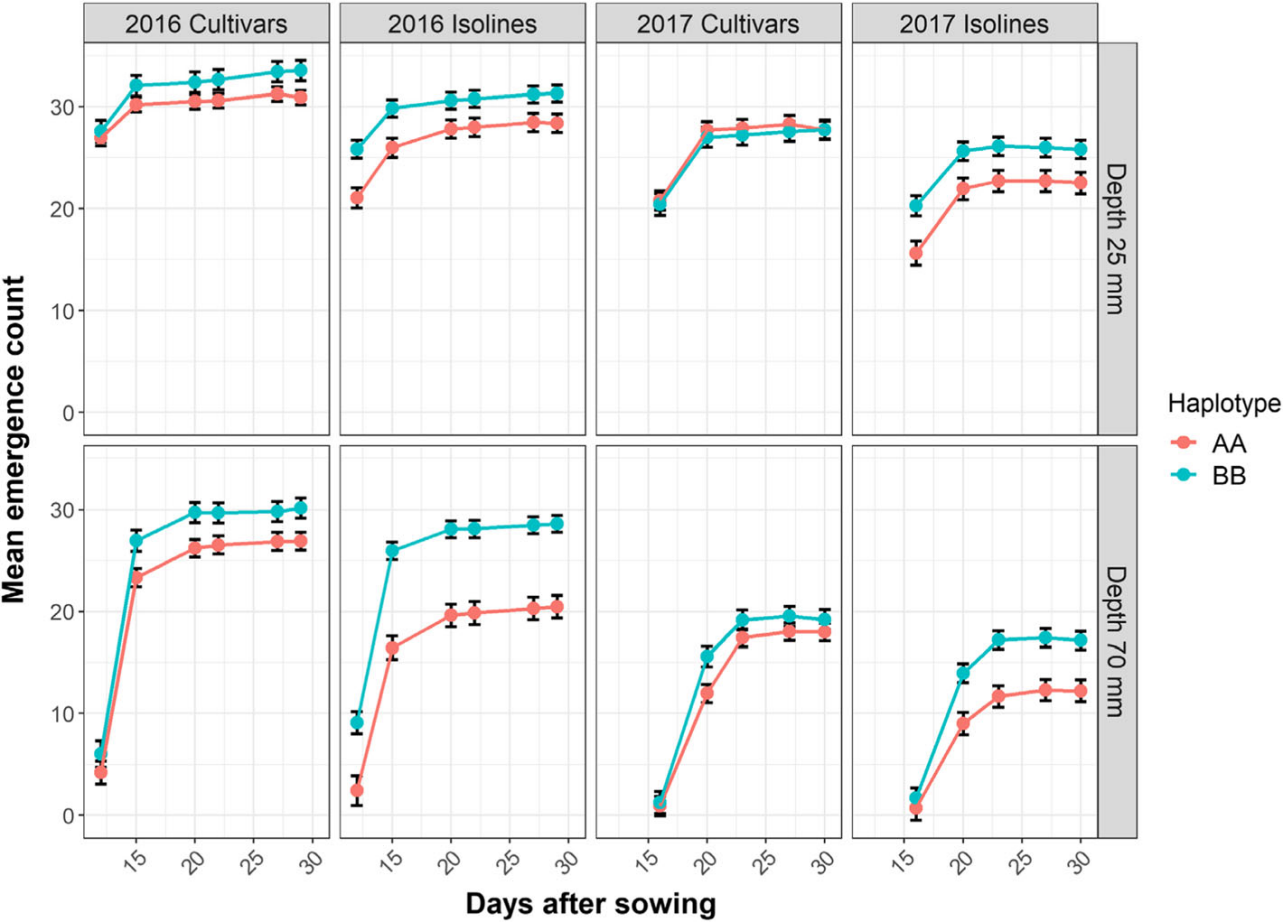
Emergence (plants per linear meter) of isolines and cultivars with long (BB) and short (AA) *Lcol-A1* alleles under normal (25 mm) and deep (70 mm) sowing in the field at Yanco in 2016 and 2017. Data are haplotype by depth by genotype class (Isolines vs Cultivars) means (obtained by averaging lines within a genotype class) ± standard error

For field validation, cultivars were sown at depths of 25 mm and 70 mm representing normal and deep sowing treatments at Yanco in western NSW (Australia) in 2016 and 2017. Emergence was counted in regular intervals during the first 5 weeks of the experiments and the mean emergence counts were plotted for both sowing depths and genotype categories (Fig. 6). In both years, more plants emerged from the deep sowing treatment in cultivars with the long *Lcol-A1* allele than in cultivars with the short allele. The difference in emergence from deep sowing was more pronounced in the Young*3/HI25M isolines, confirming the positive effect of the gene under field conditions. Interestingly, the increase in emergence afforded by *Lcol-A1* was apparent in cultivars in 2016 and in isolines in both 2016 and 2017 under the normal sowing depth as well.

### Prevalence of Lcol-A1 in an Australian diversity panel

**Fig. 7:**
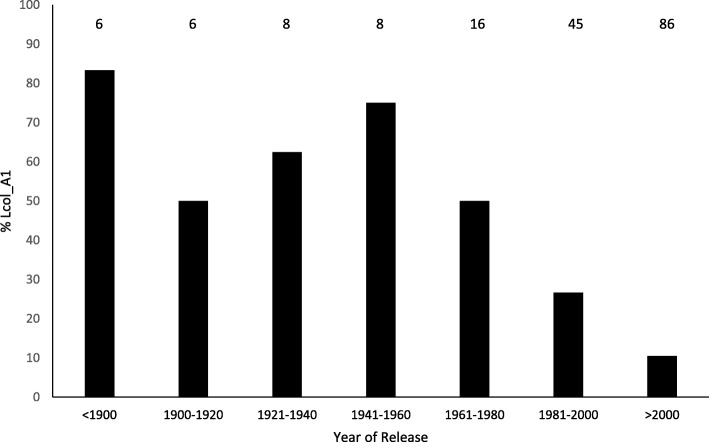
Frequency of the long *Lcol-A1* allele in an Australian diversity panel. The number of cultivars genotyped from each time period is indicated above the bars

To assess the prevalence of *Lcol-A1* in Australian germplasm, a panel of 197 cultivars and lines that were released from the year 1890 to 2015 and represent a wide range of the Australian gene pool, were screened with markers *IWB58229* and *IWA710*. Across all germplasm, the long *Lcol-A1* containing haplotype was predicted to be present in 24%, absent in 64%, and recombinant in 12% (Additional file 2: Table S2). An assessment of its evolution through the panel suggested that, despite the limited number of genotypes sampled in the early part of the twentieth century, *Lcol-1A* was reasonably common until 1960, but declined from 1960 to the latest cultivars released in 2015. To this end, the long *Lcol-A1* allele was only present in 10% of the 56 varieties released from 2000 to 2015 (Fig. 7).

## Discussion

Good crop establishment presents challenges in many wheat growing regions of the world, and long coleoptile wheats are predicted to help mitigate constraints on emergence imposed by adoption of new management practices and a changing climate. This study characterised *Lcol-A1*, a gene which promotes coleoptile length and which is present in current Australian elite wheat germplasm. The gene is responsible for coleoptile length increases even in the presence of common DELLA dwarfing genes which translated to improved emergence with deep sowing in the field. Tightly linked SNP-based markers have been developed that can be used by breeders to assist in the selection process. Information about the gene and its effect under field conditions together with validated markers delivers a useful package to breeders and the industry and represents a step change from the background-specific marker-trait associations of small effect that were often generated in QTL studies.

A Young*3/H125M population (H125M is a Halberd derivative) was chosen for fine-mapping due to the large effect of *Lcol-A1* and the clear phenotypic difference between homozygous classes. By coupling this genetic material to the fast and simple controlled environment screen used herein, we were able to delineate *Lcol-A1* to a 2 cM region, which corresponded to a physical distance of approximately 7 Mb in the RefSeqv1.0 genome sequence assembly [3]. Work is on-going to identify a candidate gene, prove its function and generate a gene-based marker for accurate selection in breeding programs. In the meantime, the flanking markers reported here will provide an efficient selection tool, particularly when flanking markers are combined to assay the Halberd-derived haplotype.

We showed that *Lcol-A1* was effective in an Espada background, conferring an approximately 25 mm increase in coleoptile length. This validation was important, as it indicated that *Lcol-A1* was effective in other genetic backgrounds additional to those used in the BSA and in the fine-mapping. Further evidence of the robustness of *Lcol-A1* was provided from previous QTL mapping studies. Rebetzke et al. [33] identified a Halberd-derived QTL for coleoptile length on 1AS, in a Cranbrook/Halberd Doubled Haploid population. In a 4-way MAGIC population, the largest effect QTL outside of the common DELLA dwarfing genes *Rht-B1* and *Rht-D1* was located on chromosome 1AS, inherited from cultivar Yitpi [35]. Yitpi and Halberd are related through pedigree, indicating that Yitpi carries *Lcol-A1* by descent. Confirmation of the effect of *Lcol-A1* in these populations provides further evidence of the utility of the gene across different genetic backgrounds.

Under conditions of deep sowing, *Lcol-A1* was associated with an increase in emergence of up to approximately 40% in tall isolines, and of approximately 15% of cultivars carrying *Rht-B1b* or *Rht-D1b*, confirming that the increase in coleoptile length afforded by *Lcol-A1* translates to improved emergence. The consistent effect of *Lcol-A1* detected in both the controlled environment screen with the field experiments confirms numerous reports about the positive relationship between coleoptile length and field emergence (e.g. [2, 42, 44]). The increased emergence under standard sowing depth with genotypes carrying *Lcol-A1* indicates that the gene may also contribute to a faster rate of coleoptile growth. Other studies also showed that increased coleoptile length is associated with increased rates of emergence [2, 38]. In Western Australia, a delay in sowing reduces yield by 28–36 kg ha^− 1^ day^− 1^ across germplasm with diverse sowing dates and phenology [39]; the increased emergence associated with *Lcol-A1* may translate to reducing these yield losses by reducing the number of later emerging seedlings. Indeed, simulation studies by Kirkegaard and Hunt [25] and Flohr et al. [19] suggest that, when combined with appropriate management strategies, the long coleoptile trait can contribute substantially to farm yield.

*Lcol-A1* is present in ~ 20% of lines that were included in the Australian wheat diversity panel. When assessing the pedigree of Halberd (long coleoptile donor used in this study), it is possible that *Lcol-A1* can be traced back to cultivars Steinwedel (a selection from the 1890’s) and Federation (released in Australia in 1901; Additional file 3: Figure S1). Although *Lcol-A1* was reasonably prevalent in Australian wheats until the 1960’s, its presence appears to have decreased to only 10% of tested cultivars released since 2000. During the 1960’s and 1970’s DELLA dwarfing genes were introduced into Australia from CIMMYT, and CIMMYT derived material became dominant parents in Australian breeding programs [8]. None of the Mexican wheats from this era which were assayed with markers carried *Lcol-A1*, and so it is possible that the decline in *Lcol-A1* prevalence is the result of breeders preferring to use parental lines from CIMMYT over the past 50–60 years [24]. Alternatively, it may be that *Lcol-A1* is linked to a trait that Australian wheat breeders are unwittingly selecting against.

Bernando [6] laments the lack of impact for breeding from the many linkage mapping studies conducted to date, but results from mapping *Lcol-A1* suggests otherwise for this trait. As opposed to novel loci from wild relatives and the challenge of reducing linkage drag when introgressing such loci, the *Lcol-A1* haplotype is present in currently available elite varieties, which should aid in its transfer within breeding programs. Current work is underway to further refine the map location and to isolate the *Lcol-A1* gene which will generate a gene-based marker. In the meantime, tightly linked and flanking SNP markers *IWB58299* and *IWA710* will be useful to predict the presence of the long allele of *Lcol-A1* in diverse germplasm.

## Conclusions

Establishment is a key phase in the life cycle of crops, and the length of the coleoptile significantly impacts upon establishment in wheat. As a result of changes in farming practices to earlier sowing into warmer soils, growers require that wheats with longer coleoptiles that are adapted to these changes are developed. Here, we confirm that *Lcol-A1* increases coleoptile length which results in improved emergence under field conditions. By developing tightly linked flanking markers, we have provided tools to allow the wheat breeding community to incorporate *Lcol-A1* more efficiently in future cultivars.

## Methods

### Phenotyping for coleoptile length – controlled environment

For coleoptile length measurements, 14 seeds/line were sown in 100 mm deep wooden trays (50:50 fertile compost/vermiculite soil mix) and grown in growth cabinets at 15 °C for 14 days (210 degree days) in the dark before coleoptile lengths were measured with a ruler.

### Bulked-segregant analysis

One-hundred and eight-eight (188) BC_1_F_2_ progeny from a Halberd*2/Chuan-Mai18 (CM18) population (Halberd is an older Australian wheat with a long coleoptile, while CM18 is a Chinese wheat with a short coleoptile [41] and 188 BC_1_F_2_ progeny from a Uruguay386*2/CM18 population (a tall Uruguay accession (AUS1517) from the Australian Winter Cereals Collection with a long coleoptile) were phenotyped for coleoptile length to identify 10 short and 10 long coleoptile lines from each population. DNA from each class was pooled separately for each population and genotyped by BSA using the 9 K SNP array at Agriculture Victoria Research, La Trobe University [10]. These populations were used for BSA only and not progressed for mapping.

### Genetic mapping of the long coleoptile gene

Two backcross populations developed from HI25M (long coleoptile Halberd derivative) and Young (short coleoptile cultivar) were used to map the long coleoptile gene. Firstly, 434 BC_1_F_2_ plants were screened with a marker derived from the *Rht-B1b* dwarfing allele [13] to identify 54 BC_1_F_2_ plants that lacked *Rht-B1b*. The elimination of the known dwarfing gene at an early stage resulted in tall progeny with only minor height variation that was derived from this population for fine mapping. These plants were genotyped with marker *IWA164* which was mapped in the 9 K SNP consensus map of wheat [10] to the 1A region identified in BSA, and BC_1_F_3_ progeny were phenotyped for coleoptile length under controlled conditions (see above). Similarly, the plants were genotyped with markers *IWA3374* and *IWA5435* from the 9 K SNP consensus map, which flanked the 1A region identified in BSA. The BC_1_ derived population was used to map the long coleoptile gene to chromosome 1A and to identify flanking SNP-based markers that were critical for the next mapping step.

To increase the resolution of the target region, BC_2_F_3_ progeny from BC_2_F_2_ plants that were heterozygous for markers in the 1A region were screened with flanking markers *IWA3374* and *IWA5435*. DNA was extracted from half-seeds of 900 BC_2_F_3_ individuals using the protocol of Ellis et al. [14]. To make the screening process more efficient, SNP-based markers were converted to Kompetitive allele specific PCR (KASP) assays [22].

Plants from half seed that carried recombination events between the flanking markers were selfed. Twelve to 16 progeny were genotyped to identify individual plants that were recombinant and homozygous in the target region. Coleoptile length was measured in homozygous progeny which were used to fine map the gene with additional 90 K SNP markers that were polymorphic between Halberd and Young, developed from the consensus map of the region.

### Validation of the 1A region

The 1A region was transferred from HI25M to Espada (an Australian cultivar) through backcrossing. BC_3_F_3_ plants were genotyped for *Rht-D1b* [13], *Rht18* [21], and the 1A locus. Lines that were homozygous wild-type for *Rht-D1*, homozygous dwarf for *Rht18*, and heterozygous for the 1A locus were selfed to produce BC_3_F_3_ derived near isogenic lines (NILs). Coleoptile length of the NILs was phenotyped using the method described above, and the same plants genotyped to assess marker-trait associations.

### Field performance

To validate the 1A locus across different genetic backgrounds and to provide evidence that the increase in coleoptile length improves emergence under field conditions, two field experiments were conducted at Yanco (34°36′53.6“S 146°24’ 54.2”E) in Southern New South Wales, Australia in 2016 and 2017. A split-plot randomised block design balanced for row and column and consisting of four replicates of 10 tall isolines (five short and five long coleoptile haplotype) of BC_1_F_5_ plants from the Young*3/H125M population as well as 11 diverse Australian cultivars (six short and five long coleoptile 1A haplotype) were sown at depths of 25 mm and 70 mm (main plots) in plots that were 7 m long and 8 rows wide, with 20 cm row spacing. Emerged plants (plants per linear meter) were counted in four rows per plot 12, 15, 20, 22, 27, and 29 days after sowing in 2016, and 16, 20, 23, 27, and 30 days after sowing in 2017. Sowing depth in the deep sowing treatment was checked by measuring the coleoptile length in seedlings after they emerged. Cultivars carried either *Rht-B1b* or *Rht-D1b* dwarfing gene and were similar in final plant height. They were chosen based on their adaptation to different climatic zones of the Australian wheat belt.

### Allele survey and pedigree assessment

To assess the frequency of the 1A haplotype from Halberd across diverse germplasm, DNA from an Australian diversity panel comprised of 197 bread wheat cultivars and lines was assayed with markers *IWB58229* and *IWA710*. To assess pedigree relationships of lines sharing a common haplotype, the Genetic Resources Information System for Wheat and Triticale (GRIS; wheatpedigree.net) was queried.

### Statistical analyses

The controlled environment and the 2016 and 2017 field experiment datasets were analysed using the linear mixed model software asreml [9] for R (R [29]).

For the controlled environment experiment, cultivar and isoline genotype class groups were analysed separately. To compare haplotypes, the traditional ‘one-way ANOVA’ analysis for comparing two treatment groups was extended to a linear mixed model by representing varieties comprising the particular genotype class as a random effects factor in the statistical model, thus ensuring the correct degrees of freedom were used for the haplotype comparison. F-tests for the significance of the haplotype term yield the *p*-values for the comparison between AA and BB haplotypes within each genotype class. Potential outliers were identified using the 1.5x interquartile range criterion [4].

The field experiments in 2016 and 2017 were analysed separately, although isolines and cultivars were analysed together within each trial. The field experiments involve factorial treatment structures for haplotype, genotype class and sowing depth, modelled as fixed effects. Since the haplotype/genotype class combinations represent groups of varieties, variety was included in the model as a nested random effects factor, and variety interaction terms were therefore modelled as random effects terms. Further random effects terms were included to represent the spatial structure of the trial layout, namely block, plot and subplot (representing row within plot). The models incorporated the recording of observations across multiple dates by accounting for autocorrelation structure across the (unevenly spaced) dates, and allowing heterogeneity of the residual variances across dates. For the 2016 dataset the model also allowed for unequal variances that were observed between the two sowing depths.

## Additional files

Additional file 1: **Table S1.** All significant marker-trait associations detected from BSA using the 9 K SNP array in the Halberd*2/CM18 and Uruguay*2/CM18 populations. (DOCX 29 kb)

Additional file 2: **Table S2.** Genotyping results from the Australian diversity panel. Cultivar, year of release, and allele call (A indicates short allele; B indicates long allele) for markers IWB58229 and IWA710 are shown. (DOCX 31 kb)

Additional file 3: **Figure S1.** Pedigree of Halberd. Cultivars which were genotyped in the diversity panel carrying the long (green) or short (yellow) *Lcol-A1* alleles are highlighted. Gaza, a cultivar carrying a recombinant haplotype is highlighted in orange. (DOCX 270 kb)

## Data Availability

The datasets used and/or analysed during the current study are available from the corresponding author on reasonable request.

## Abbreviations

AFLP: Amplified fragment length polymorphism
BSA: Bulked-segregant analysis
GWAS: Genome wide association study
KASP: Kompetitive allele specific PCR
MAGIC: Multi-parent advanced generation intercross
MTA: Marker trait association
QTL: Quantitative trait locus
SNP: Single nucleotide polymorphism

## References

[1] Allan RE (1980). Influence of semidwarfism and genetic background on stand establishment of wheat. Crop Sci.

[2] Allan RE, Vogel OA, Peterson CJ (1962). Seedling emergence rate of fall-sown wheat and its association with plant height and coleoptile length. Agron J.

[3] Appels R, The International Wheat Genome Sequencing Initiative (IWGSC). Shifting the limits in wheat research and breeding using a fully annotated reference genome. Science. 2018;345:1251788. 10.1126/science.aar7191 30115783

[4] Barbato G, Barini EM, Genta G, Levi R (2011). Features and performance of some outlier detection methods. J Appl Stat.

[5] Beharav A, Cahaner A, Pinthus MJ (1998). Genetic correlations between culm length, grain yield and seedling elongation within tall (*rht1*) and semi-dwarf (*Rht1*) spring wheat (*Triticum aestivum* L.). Eur J Agron.

[6] Bernardo R (2016). Bandwagons I, too, have known. Theor Appl Genet.

[7] Bhatt GM, Sheedi SM (1986). Sensitivity of wheat coleoptile to variation in temperature. Cereal Res Commun.

[8] Brennan JP, Quade KJ (2004). Analysis of the impact of CIMMYT research on the Australian wheat industry.

[9] Butler DG. 2009. Asreml: asreml() fits the linear mixed model. *R package version 3.0*. http://www.vsni.co.uk.

[10] Cavanagh CR, Chao S, Wang S, Huang BE, Stephen S, Kiani S, Forrest K, Saintenac C, Brown-Guedira GL, Akhunova A, See D, Bai G, Pumphrey M, Tomar L, Wong D, Kong S, Reynolds M, da Silva ML, Bockelman H, Talbert L, Anderson JA, Dreisigacker S, Baenziger S, Carter A, Korzun V, Morrell PL, Dubcovsky J, Morell MK, Sorrells ME, Hayden MJ, Akhunov E (2013). Genome-wide comparative diversity uncovers multiple targets of selection for improvement in hexaploid wheat landraces and cultivars. Proc Natl Acad Sci.

[11] Collis-George N, Yoganathan P (1985). The effect of soil strength on germination and emergence of wheat (*Triticum aestivum* L.). I. Low shear strength conditions. Soil Research.

[12] Collis-George N, Yoganathan P (1985). The effect of soil strength on germination and emergence of wheat (*Triticum aestivum* L.). II. High shear strength conditions. Soil Research.

[13] Ellis M, Spielmeyer W, Gale K, Rebetzke G, Richards R (2002). "perfect" markers for the Rht-B1b and Rht-D1b dwarfing genes in wheat. Theor Appl Genet.

[14] Ellis MH, Rebetzke GJ, Azanza F, Richards RA, Spielmeyer W (2005). Molecular mapping of gibberellin-responsive dwarfing genes in bread wheat. Theor Appl Genet.

[15] Feather J, Qualset C, Vogt H (1968). Planting depth critical for short-statured wheat varieties. Calif Agric.

[16] Fletcher A, Lawes R, Weeks C (2016). Crop area increases drive earlier and dry sowing in Western Australia: implications for farming systems. Crop Pasture Sci.

[17] Fletcher AL, Robertson MJ, Abrecht DG, Sharma DL, Holzworth DP (2015). Dry sowing increases farm level wheat yields but not production risks in a Mediterranean environment. Agric Syst.

[18] Flohr BM, Hunt JR, Kirkegaard JA, Evans JR (2017). Water and temperature stress define the optimal flowering period for wheat in South-Eastern Australia. Field Crop Res.

[19] Flohr BM, Hunt JR, Kirkegaard JA, Evans JR, Lilley JM (2018). Genotype × management strategies to stabilise the flowering time of wheat in the south-eastern Australian wheatbelt. Crop Pasture Sci.

[20] Flohr BM, Hunt JR, Kirkegaard JA, Evans JR, Trevaskis B, Zwart A, Swan A, Fletcher AL, Rheinheimer B (2018). Fast winter wheat phenology can stabilise flowering date and maximise grain yield in semi-arid Mediterranean and temperate environments. Field Crop Res.

[21] Ford BA, Foo E, Sharwood R, Karafiatova M, Vrána J, MacMillan C, Nichols DS, Steuernagel B, Uauy C, Doležel J, Chandler PM, Spielmeyer W (2018). *Rht18* semidwarfism in wheat is due to increased *GA 2-oxidaseA9* expression and reduced GA content. Plant Physiol.

[22] He C, Holme J, Anthony J, Fleury D, Whitford R (2014). SNP genotyping: the KASP assay. Crop breeding: methods and protocols.

[23] Hedden P (2003). The genes of the green revolution. Trends Genet.

[24] Joukhadar R, Daetwyler HD, Bansal UK, Gendall AR, Hayden MJ. Genetic diversity, population structure and ancestral origin of Australian wheat. Front Plant Sci. 2017;8:2115. 10.3389/fpls.2017.02115 PMC5733070 29312381

[25] Kirkegaard JA, Hunt JR (2010). Increasing productivity by matching farming system management and genotype in water-limited environments. J Exp Bot.

[26] Li G, Bai G, Carver BF, Elliott NC, Bennett RS, Wu Y, Hunger R, Bonman JM, Xu X (2017). Genome-wide association study reveals genetic architecture of coleoptile length in wheat. Theor Appl Genet.

[27] Li P, Chen J, Wu P, Zhang J, Chu C, See D, Brown-Guedira G, Zemetra R, Souza E (2011). Quantitative trait loci analysis for the effect of Rht-B1 dwarfing gene on coleoptile length and seedling root length and number of bread wheat. Crop Sci.

[28] Lilley JM, Kirkegaard JA (2016). Farming system context drives the value of deep wheat roots in semi-arid environments. J Exp Bot.

[29] Core Team R. R: a language and environment for statistical computing. Vienna: R Foundation for Statistical Computing; 2017. https://www.r-project.org/

[30] Radford B (1987). Effect of constant and fluctuating temperature regimes and seed source on the coleoptile length of tall and semidwarf wheats. Aust J Exp Agric.

[31] Rebetzke GJ, Appels R, Morrison AD, Richards RA, McDonald G, Ellis MH, Spielmeyer W, Bonnett DG (2001). Quantitative trait loci on chromosome 4B for coleoptile length and early vigour in wheat (*Triticum aestivum* L.). Aust J Agric Res.

[32] Rebetzke GJ, Bruce SE, Kirkegaard JA (2005). Longer coleoptiles improve emergence through crop residues to increase seedling number and biomass in wheat (*Triticum aestivum* L.). Plant Soil.

[33] Rebetzke GJ, Ellis MH, Bonnett DG, Richards RA (2007). Molecular mapping of genes for coleoptile growth in bread wheat (*Triticum aestivum* L.). Theor Appl Genet.

[34] Rebetzke GJ, Richards RA, Sirault XRR, Morrison AD (2004). Genetic analysis of coleoptile length and diameter in wheat. Aust J Agric Res.

[35] Rebetzke GJ, Verbyla AP, Verbyla KL, Morell MK, Cavanagh CR (2014). Use of a large multiparent wheat mapping population in genomic dissection of coleoptile and seedling growth. Plant Biotechnol J.

[36] Rebetzke GJ, Zheng B, Chapman SC (2016). Do wheat breeders have suitable genetic variation to overcome short coleoptiles and poor establishment in the warmer soils of future climates?. Funct Plant Biol.

[37] Sayre KD, Rajaram S, Fischer RA (1997). Yield potential progress in short bread wheats in Northwest Mexico. Crop Sci.

[38] Schillinger WF, Donaldson E, Allan RE, Jones SS (1998). Winter wheat seedling emergence from deep sowing depths. Agron J.

[39] Shackley B, Anderson W (1995). Responses of wheat cultivars to time of sowing in the southern wheatbelt of Western Australia. Aust J Exp Agric.

[40] Singh K, Shukla S, Kadam S, Semwal VK, Singh NK, Khanna-Chopra R (2015). Genomic regions and underlying candidate genes associated with coleoptile length under deep sowing conditions in a wheat RIL population. J Plant Biochem Biotechnol.

[41] Spielmeyer W, Hyles J, Joaquim P, Azanza F, Bonnett D, Ellis ME, Moore C, Richards RA (2007). A QTL on chromosome 6A in bread wheat (Triticum aestivum) is associated with longer coleoptiles, greater seedling vigour and final plant height. Theor Appl Genet.

[42] Sunderman DW (1964). Seedling emergence of winter wheats and its association with depth of sowing, coleoptile length under various conditions, and plant height. Agron J.

[43] Wang S, Wong D, Forrest K, Allen A, Chao S, Huang BE, Maccaferri M, Salvi S, Milner SG, Cattivelli L, Mastrangelo AM, Whan A, Stephen S, Barker G, Wieseke R, Plieske J, Lillemo M, Mather D, Appels R, Dolferus R, Brown-Guedira G, Korol A, Akhunova AR, Feuillet C, Salse J, Morgante M, Pozniak C, Luo M-C, Dvorak J, Morell M, Dubcovsky J, Ganal M, Tuberosa R, Lawley C, Mikoulitch I, Cavanagh C, Edwards KJ, Hayden M, Akhunov E, International Wheat Genome Sequencing C (2014). Characterization of polyploid wheat genomic diversity using a high-density 90 000 single nucleotide polymorphism array. Plant Biotechnol J.

[44] Whan B (1976). The emergence of semidwarf and standard wheats, and its association with coleoptile length. Aust J Exp Agric.

[45] Yu J-B, Bai G-H (2010). Mapping quantitative trait loci for long coleoptile in Chinese wheat landrace Wangshuibai. Crop Sci.

