# Supplementary material for: Increase in coleoptile length and establishment by Lcol-A1, a genetic locus with major effect in wheat
Source: BMC Plant Biol. 2019 Jul 29;19:332. doi: 10.1186/s12870-019-1919-3 (PMC6664495; doi:10.1186/s12870-019-1919-3)
Supplement: Supplementary file 1 — Table S1. All significant marker-trait associations detected from BSA using the 9 K SNP array in the Halberd*2/CM18 and Uruguay*2/CM18 populations. (DOCX 29 kb) [file 12870_2019_1919_MOESM1_ESM.docx]

Additional file 1: **Table S1. All significant marker-trait associations detected from BSA using the 9K SNP array in the Halberd*2/CM18 and Uruguay*2/CM18 populations.**

| SNP Name | SNP ID | 9K_Chr | 9K_Loc | 90K_Chr | 90K_Loc |
| --- | --- | --- | --- | --- | --- |
| **Halberd/CM18** | | | | | |
| wsnp_Ku_c17726_26872129 | IWA6636 | 1A | 51.12 | 1A | 71.05 |
| wsnp_Ex_c3253_5995011 | IWA3399 | 1A | 54.28 | NA |  |
| wsnp_Ex_c6826_11774795 | IWA4578 | 1A | 57.95 | 1A | 70.10 |
| wsnp_Ku_c3468_6420199 | IWA6942 | 1A | 57.95 | 1A | 70.10 |
| wsnp_Ex_c3747_6824863 | IWA3612 | 1A | 57.95 | 1A | 70.10 |
| wsnp_Ex_c8364_14095508 | IWA4797 | 1A | 62.39 | 1A | 71.10 |
| wsnp_Ku_c37925_46679146 | IWA6972 | 1A | 70.70 | 1A | 71.10 |
| wsnp_Ku_c33917_43336069 | IWA6934 | 1A | 109.32 | 1A | 101.19 |
| wsnp_Ex_c18733_27607958 | IWA2345 | 1B | 27.36 | 1B | 62.58 |
| wsnp_BE444846B_Ta_2_1 | IWA158 | 1B | 30.47 | NA |  |
| wsnp_BE495786B_Ta_2_1 | IWA269 | 1B | 60.25 | NA |  |
| wsnp_Ex_c10084_16572374 | IWA1275 | 2A | 158.93 | 2A | 123.47 |
| wsnp_Ku_c4507_8157580 | IWA7076 | 2B | 92.63 | 2B | 92.57 |
| wsnp_BF202681B_Ta_2_2 | IWA429 | 2B | 94.01 | 2B | 92.57 |
| wsnp_BE399688B_Ta_2_1 | IWA10 | 2B | 110.85 | 2B | 93.47 |
| wsnp_Ku_c12517_20191465 | IWA6462 | 2B | 110.85 | 2B | 96.99 |
| wsnp_Ex_rep_c106085_90293854 | IWA5149 | 2B | 114.69 | 2B | 97.26 |
| wsnp_BE445278B_Ta_2_3 | IWA170 | 2B | 126.28 | 2B | 96.99 |
| wsnp_CAP11_c5474_2542512 | IWA776 | 2B | 126.28 | 2B | 96.99 |
| wsnp_BE445278B_Ta_2_1 | IWA169 | 2B | 126.28 | 2B | 96.99 |
| wsnp_Ex_c58019_59494143 | IWA4354 | 2D | 10.18 | NA |  |
| wsnp_Ex_c10014_16477392 | IWA1270 | 3A | 2.48 | NA |  |
| wsnp_Ex_c6833_11783076 | IWA4582 | 3A | 3.93 | NA |  |
| wsnp_Ku_c217_430915 | IWA6716 | 3A | 166.34 | NA |  |
| wsnp_Ex_c26213_35462685 | IWA3031 | 3A | 171.83 | NA |  |
| wsnp_Ex_c33012_41567026 | IWA3422 | 4A | 12.39 | 4A | 152.99 |
| wsnp_Ex_c16814_25373602 | IWA2170 | 4A | 43.79 | NA |  |
| wsnp_Ku_c2317_4448102 | IWA6733 | 4A | 103.03 | 4A | 68.28 |
| wsnp_BJ224975A_Ta_2_1 | IWA589 | 5A | 66.23 | NA |  |
| wsnp_Ex_rep_c66689_65010988 | IWA5287 | 5A | 66.67 | 5A | 91.30 |
| wsnp_Ex_c7729_13177883 | IWA4719 | 5A | 69.07 | 5A | 89.96 |
| wsnp_Ex_c59520_60358626 | IWA4391 | 5A | 78.14 | 5A | 84.13 |
| wsnp_Ex_c47684_52820187 | IWA3996 | 5A | 87.89 | 5A | 81.13 |
| wsnp_Ex_c18941_27840714 | IWA2363 | 5A | 94.73 | 5A | 74.78 |
| wsnp_CAP11_c1740_947838 | IWA687 | 5A | 98.81 | 5A | 68.95 |
| wsnp_Ex_c12678_20148981 | IWA1669 | 5A | 98.81 | NA |  |
| wsnp_CAP8_c606_443906 | IWA1201 | 5A | 98.81 | NA |  |
| wsnp_CAP8_rep_c5486_2606556 | IWA1236 | 5A | 107.73 | NA |  |
| wsnp_Ra_c13424_21239985 | IWA7608 | 5B | 182.27 | 5B | 43.56 |
| wsnp_Ex_c34597_42879693 | IWA3487 | 6A | 180.19 | 6A | 125.45 |
| wsnp_Ex_c749_1472258 | IWA4691 | 6A | 201.95 | 6A | 138.04 |
| wsnp_Ex_c15708_24056750 | IWA2054 | 6A | 206.03 | NA |  |
| wsnp_Ex_c4815_8597139 | IWA4011 | 6B | 21.76 | 6B | 33.75 |
| wsnp_Ex_c4815_8597064 | IWA4010 | 6B | 21.76 | 6B | 33.75 |
| wsnp_Ex_c21068_30195276 | IWA2569 | 7A | 65.82 | 7A | 135.81 |
| wsnp_Ex_c10094_16590746 | IWA1278 | 7A | 79.92 | NA |  |
| wsnp_Ex_c6348_11045240 | IWA4482 | 7A | 80.62 | NA |  |
| wsnp_JD_c14118_13933380 | IWA5844 | 7A | 82.34 | 7A | 126.80 |
| wsnp_JD_c7987_9038994 | IWA6183 | 7A | 82.34 | 7A | 135.81 |
| wsnp_Ex_c4883_8705816 | IWA4037 | 7A | 96.21 | 7A | 135.54 |
| wsnp_CAP11_c2211_1157166 | IWA707 | 7A | 103.71 | 7A | 135.81 |
| wsnp_Ex_c27914_37074773 | IWA3129 | 7B | 45.25 | 7B | 67.47 |
| wsnp_Ex_c5925_10397213 | IWA4380 | 7B | 48.48 | 7B | 69.93 |
| wsnp_Ex_c6797_11732168 | IWA4574 | 7B | 51.85 | 7B | 71.66 |
| wsnp_Ex_c12556_19992321 | IWA1654 | 7B | 164.21 | 7B | 162.53 |
| wsnp_Ex_c49035_53760034 | IWA4046 | NA |  | NA |  |
| wsnp_JD_c5757_6915127 | IWA6110 | NA |  | NA |  |
| wsnp_CAP7_c5487_2464794 | IWA1096 | NA |  | NA |  |
| **Uruguay/CM18** | | | | | |
| wsnp_Ex_c5323_9408829 | IWA4163 | 1A | 35.82 | 1A | 51.09 |
| wsnp_Ku_c11896_19337444 | IWA6441 | 1A | 39.00 | NA |  |
| wsnp_BE586140A_Ta_2_1 | IWA360 | 1A | 40.65 | 1A | 55.18 |
| wsnp_Ex_c34821_43076533 | IWA3499 | 1A | 51.12 | 1A | 71.48 |
| wsnp_Ex_c3253_5995011 | IWA3339 | 1A | 54.28 | NA |  |
| wsnp_Ex_c2749_5091813 | IWA3115 | 1A | 56.97 | 1A | 70.10 |
| wsnp_JD_rep_c49359_33578909 | IWA6260.1 | 1A | 57.95 | 1A | 70.10 |
| wsnp_Ex_rep_c104050_88861052 | IWA5109 | 1A | 57.95 | 1A | 70.10 |
| wsnp_Ra_c26956_36503468 | IWA7804 | 1A | 57.95 | 1A | 70.10 |
| wsnp_Ex_c2389_4477096 | IWA2847 | 1A | 57.95 | 1A | 70.10 |
| wsnp_Ex_c3142_5808330 | IWA3338 | 1A | 64.39 | 1A | 70.10 |
| wsnp_BE518393A_Td_2_3 | IWA352 | 1A | 64.39 | 1A | 70.10 |
| wsnp_Ex_rep_c66382_64577768 | IWA5226 | 1A | 67.01 | 1A | 70.10 |
| wsnp_CAP11_c1029_611774 | IWA639 | 1A | 68.32 | 1A | 70.10 |
| wsnp_Ku_rep_c71909_71634013 | IWA7505 | 1A | 68.65 | 1A | 70.79 |
| wsnp_Ex_c4204_7594348 | IWA3809 | 2A | 214.43 | NA |  |
| wsnp_Ex_c7776_13247365 | IWA4723 | 2B | 4.98 | 2B | 20.86 |
| wsnp_Ex_c16144_24583060 | IWA2104.1 | 2B | 34.84 | 2B | 134.46 |
| wsnp_Ex_c2445_4573233 | IWA2903 | 2B | 169.50 | 2B | 109.53 |
| wsnp_Ra_c21559_30963208 | IWA7748 | 3B | 66.39 | NA |  |
| wsnp_Ra_c9123_15295482 | IWA8096 | 4A | 18.44 | NA |  |
| wsnp_CAP12_rep_c4278_1949802 | IWA1006 | 4B | 68.73 | 4B | 71.46 |
| wsnp_Ex_c12684_20157261 | IWA1670 | 5A | 20.17 | 5A | 117.67 |
| wsnp_Ku_c792_1636348 | IWA7306 | 7A | 6.21 | 7A | 42.08 |
| wsnp_CAP12_c3056_1439567 | IWA954 | 7A | 32.82 | 7A | 80.36 |
| wsnp_Ex_c7030_12111917 | IWA4614 | 7A | 34.11 | 7A | 82.95 |
| wsnp_Ex_c351_689415 | IWA3513 | 7B | 139.18 |  | 143.23 |
| wsnp_JD_c15748_15112101 | IWA5866 | NA |  | NA |  |
