# Supplementary material for: Increase in coleoptile length and establishment by Lcol-A1, a genetic locus with major effect in wheat
Source: BMC Plant Biol. 2019 Jul 29;19:332. doi: 10.1186/s12870-019-1919-3 (PMC6664495; doi:10.1186/s12870-019-1919-3)
Supplement: Supplementary file 2 — Table S2. Genotyping results from the Australian diversity panel. Cultivar, year of release, and allele call (A indicates short allele; B indicates long allele) for markers IWB58229 and IWA710 are shown. (DOCX 31 kb) [file 12870_2019_1919_MOESM2_ESM.docx]

Additional file 2: **Table S2. Genotyping results from the Australian diversity panel.** Cultivar, year of release, and allele call (A indicates short allele; B indicates long allele) for markers IWB58229 and IWA710 are shown.

| **Cultivar** | **Year** | **IWB58229** | **IWA710** |
| --- | --- | --- | --- |
| AGT_KATANA | 2009 | A | A |
| AGT_SCYTHE | 2005 | A | A |
| ANNUELLO | 2001 | A | A |
| AROONA | 1981 | B | A |
| ARRINO | 1997 | B | B |
| AXE | 2007 | A | A |
| BARHAM | 2006 | A | B |
| BATAVIA | 1991 | A | A |
| BAXTER | 1998 | A | A |
| BEAUFORT | 2008 | A | A |
| BENCUBBIN | 1929 | B | B |
| BINNU | 2006 | B | B |
| BOLAC | 2006 | A | A |
| BOUNTY | 2008 | A | A |
| BOWIE | 1997 | B | A |
| BRAEWOOD | 2001 | A | A |
| BRENNAN | 1998 | A | A |
| BROOKTON | 1997 | A | A |
| BT_SCHOMBURGK | 1994 | A | B |
| BULLARING | 2006 | A | B |
| BUMPER | 2008 | A | A |
| CADOUX | 1992 | B | B |
| CALINGIRI | 1997 | A | A |
| CARINYA | 2008 | A | A |
| CARNAMAH | 1996 | A | A |
| CASCADES | 1994 | B | A |
| CATALINA | 2007 | A | A |
| CHARA | 1998 | A | A |
| CHEYENNE | 1933 | A | A |
| CLEARFIELD_WHT_JNZ | 2001 | A | A |
| CLEARFIELD_WHT_STL | 2005 | A | A |
| COBRA | 2011 | A | A |
| CONDOR | 1973 | A | A |
| COOK | 1977 | A | A |
| CORACK | 2011 | A | A |
| CORRELL | 2006 | B | B |
| CRANBROOK | 1985 | A | A |
| CRUSADER | 2008 | A | A |
| CUNNINGHAM | 1990 | A | A |
| CURRAWA | 1912 | B | B |
| DAKOTA | 2008 | A | A |
| DART | 2010 | A | A |
| DERRIMUT | 2006 | A | A |
| DIAMONDBIRD | 1997 | A | A |
| DIRK | 1951 | B | B |
| DRYSDALE | 2001 | A | A |
| DUNDEE | 1927 | B | B |
| EAGLE | 1965 | B | B |
| EGA_BONNIE_ROCK | 2002 | B | A |
| EGA_BURKE | 2006 | A | A |
| EGA_CASTLE_ROCK | 2004 | A | A |
| EGA_EAGLE_ROCK | 2004 | B | B |
| EGA_EAGLEHAWK | 2006 | A | A |
| EGA_GREGORY | 2004 | A | A |
| EGA_JITARNING | 2004 | B | A |
| EGA_KIDMAN | 2008 | A | A |
| EGA_WEDGETAIL | 2002 | A | A |
| EGA_WENTWORTH | 2004 | A | A |
| EGA_WYLIE | 2004 | A | A |
| EGRET | 1973 | A | A |
| EINSTEIN | 2007 | A | A |
| ELLISON | 2003 | A | A |
| ELMORE_CL_PLUS | 2012 | A | A |
| ENDURE | 2008 | A | A |
| ESPADA | 2008 | A | A |
| ESTOC | 2010 | A | A |
| EXCALIBUR | 1990 | B | B |
| FANG | 2008 | A | A |
| FEDERATION | 1901 | B | B |
| FELIX | 1917 | A | A |
| FESTIGUAY | 1963 | B | B |
| FORD | 1916 | A | A |
| FORREST | 2010 | A | A |
| FORTUNE | 1916 | A | A |
| FRAME | 1997 | B | B |
| FREE_GALLIPOLI | 1925 | A | A |
| FRONTANA | 1930 | A | A |
| GABO | 1942 | B | B |
| GAUNTLET | 2011 | A | A |
| GAZA | 1973 | B | A |
| GAZELLE | 2009 | A | B |
| GBA_COMBAT | 2003 | A | A |
| GBA_HUNTER | 2005 | A | A |
| GBA_RUBY | 2003 | A | A |
| GBA_SAPPHIRE | 2004 | A | A |
| GHURKA | 1924 | B | B |
| GILES | 1999 | A | A |
| GLADIUS | 2007 | A | A |
| GLOVER | 2001 | A | A |
| GRENADE_CL_PLUS | 2012 | A | A |
| GUARDIAN | 2007 | A | A |
| GULAR | 1927 | B | B |
| HALBERD | 1969 | B | B |
| HARPER | 1899 | B | B |
| HARRISMITH | 2001 | B | A |
| HERON | 1958 | B | B |
| HUDSONS_EARLY_PURPLE_STRAW | 1890 | B | B |
| IMPALA | 2011 | A | A |
| IMPOSE_CL_PLUS | 2011 | A | A |
| INSIGNIA | 1946 | B | B |
| JANZ | 1989 | A | A |
| JUSTICA_CL_PLUS | 2010 | A | A |
| KELALAC | 1988 | A | A |
| KENNEDY | 1998 | A | A |
| KING_ROCK | 2009 | A | B |
| KITE | 1973 | B | B |
| KORD_CL_PLUS | 2010 | A | A |
| KRICHAUFF | 1998 | B | A |
| KUKRI | 1999 | A | A |
| KUNJIN | 2010 | B | B |
| LANCE | 1978 | B | A |
| LANG | 2000 | A | A |
| LINCOLN | 2007 | A | A |
| LORIKEET | 2001 | A | A |
| MACE | 2008 | A | A |
| MACHETE | 1985 | B | B |
| MACKELLAR | 2002 | A | A |
| MADDEN | 1973 | B | B |
| MAGENTA | 2007 | B | B |
| MANSFIELD | 2010 | A | B |
| MAROMBI | 2001 | A | A |
| MEERING | 1984 | A | A |
| MERINDA | 2008 | A | A |
| MILLEWA | 1979 | A | B |
| MOLINEUX | 1988 | A | A |
| NABAWA | 1915 | B | B |
| NAPAROO | 2008 | A | A |
| OLYMPIC | 1956 | B | B |
| OXLEY | 1974 | A | A |
| PEAKE | 2007 | A | A |
| PELSART | 1994 | A | A |
| PETREL | 1996 | A | B |
| PHANTOM | 2010 | B | B |
| PITIC_62 | 1962 | A | A |
| PRESTON | 2008 | A | B |
| PUGSLEY | 2002 | B | B |
| PURPLE_STRAW | 1860 | A | A |
| QALBIS | 2002 | B | A |
| RANEE | 1924 | B | B |
| RAVEN | 1963 | B | B |
| REES | 2003 | A | A |
| REVENUE | 2009 | A | A |
| ROSELLA | 1985 | A | A |
| RUDD | 2002 | A | A |
| SABRE | 1952 | B | B |
| SCOUT | 2009 | B | B |
| SENTINEL | 2005 | A | A |
| SERI_82 | 1982 | A | A |
| SHIELD | 2012 | A | A |
| SILVERSTAR | 1998 | A | A |
| SONORA_64 | 1964 | A | A |
| SPEAR | 1983 | B | B |
| SPITFIRE | 2011 | A | A |
| STAMPEDE | 2007 | A | A |
| STEINWEDEL | 1890 | B | B |
| STILETTO | 1993 | B | B |
| STRZELECKI | 2000 | A | A |
| SUNBRI | 1990 | A | A |
| SUNELG | 1984 | B | B |
| SUNGUARD | 2011 | A | A |
| SUNLIN | 1996 | B | B |
| SUNSOFT_98 | 1998 | A | A |
| SUNSTATE | 1993 | A | A |
| SUNTOP | 2011 | A | A |
| SUNVALE | 1994 | A | A |
| SUNVEX | 2008 | B | A |
| SUNZELL | 2007 | A | A |
| TAMMARIN_ROCK | 2005 | A | A |
| TAMMIN | 1994 | B | B |
| TASMAN | 1993 | A | A |
| TENNANT | 1998 | A | A |
| TIMGALEN | 1967 | B | B |
| TINCURRIN | 1978 | B | B |
| TRIDENT | 1993 | B | B |
| VENTURA | 2004 | A | A |
| VERANOPOLIS | 1950 | A | A |
| WAAGAN | 2009 | A | A |
| WALLUP | 2011 | A | A |
| WARDS_PROLIFIC | 1890 | B | B |
| WARIGAL | 1978 | B | A |
| WESTONIA | 1997 | A | A |
| WHISTLER | 1998 | A | A |
| WILLS | 2008 | A | A |
| WORRAKATTA | 1997 | B | A |
| WW15 | 1969 | A | A |
| WYALKATCHEM | 2001 | A | A |
| WYLAH | 1999 | A | A |
| YAKTANA_54 | 1954 | A | A |
| YANDANOOKA | 2008 | A | A |
| YANDILLA | 1899 | B | B |
| YAWA | 2012 | B | B |
| YECORA_70 | 1970 | A | A |
| YENDA | 2006 | A | B |
| YITPI | 2000 | B | B |
| YOUNG | 2005 | A | A |
| ZEBU | 2008 | A | A |
| ZIPPY | 2009 | A | A |
