# Supplementary material for: Increase in coleoptile length and establishment by Lcol-A1, a genetic locus with major effect in wheat
Source: BMC Plant Biol. 2019 Jul 29;19:332. doi: 10.1186/s12870-019-1919-3 (PMC6664495; doi:10.1186/s12870-019-1919-3)
Supplement: Supplementary file 3 — Figure S1. Pedigree of Halberd. Cultivars which were genotyped in the diversity panel carrying the long (green) or short (yellow) Lcol-A1 alleles are highlighted. Gaza, a cultivar carrying a recombinant haplotype is highlighted in orange. (DOCX 270 kb) [file 12870_2019_1919_MOESM3_ESM.docx]

Additional file 3: **Figure S1. Pedigree of Halberd.** Cultivars which were genotyped in the diversity panel carrying the long (green) or short (yellow) *Lcol-A1* alleles are highlighted. Gaza, a cultivar carrying a recombinant haplotype is highlighted in orange.
